# European Clinical Neuropsychology: Role in Healthcare and Access to Neuropsychological Services

**DOI:** 10.3390/healthcare9060734

**Published:** 2021-06-15

**Authors:** Erich Kasten, Fernando Barbosa, Mary H. Kosmidis, Bengt A. Persson, Marios Constantinou, Gus A. Baker, Sandra Lettner, Laura Hokkanen, Amélie Ponchel, Sara Mondini, Maria K. Jonsdottir, Nataliya Varako, Tomas Nikolai, Aiste Pranckeviciene, Lauren Harper, Erik Hessen

**Affiliations:** 1 Department of Psychology, MSH University of Applied Sciences & Medical University, D-20457 Hamburg, Germany; 2 Laboratory of Neuropsychophysiology, Faculty of Psychology and Education Sciences, University of Porto, 4099-002 Porto, Portugal; fernandobarbosa@me.com; 3 Lab of Cognitive Neuroscience, School of Psychology, Aristotle University of Thessaloniki, 541 24 Thessaloniki, Greece; mary.h.kosmidis@gmail.com; 4 Department of Psychology, Linnaeus University, 351 95 Växjö, Sweden; bengt.persson@blixtmail.se; 5 Department of Social Sciences, School of Humanities and Social Sciences, University of Nicosia, 2417 Nicosia, Cyprus; drconstantinou@gmail.com; 6 Clinical Neuropsychology-Molecular and Clinical Pharmacology, University of Liverpool, Liverpool L69 3BX, UK; gus@liverpool.ac.uk; 7 Association for Neuropsychology Austria, 4921 Bad Häring, Austria; Sandra.Lettner@bhs.at; 8 Department of Psychology and Logopedics, Faculty of Medicine, University of Helsinki, 00100 Helsinki, Finland; laura.hokkanen@helsinki.fi; 9 GHU Paris Psychiatry & Neurosciences, 75014 Paris, France; amelie.ponchel@gmail.com; 10 Department of Philosophy, Sociology, Pedagogy and Applied Psychology, University of Padua, 35122 Padua, Italy; sara.mondini@unipd.it; 11 Department of Psychology, Reykjavik University, 101 Reykjavik, Iceland; mariakj@ru.is; 12 Psychological Methodology Department, Faculty of Psychology, Lomonosov Moscow State University, 119991 Moscow, Russia; nvarako@mail.ru; 13 Research Center of Neurology, 125367 Moscow, Russia; 14 Department of Neurology, Charles University in Prague, 110 00 Prague, Czech Republic; tomas.nikolai@lf1.cuni.cz; 15 Department of Health Psychology, Medical Academy, Lithuanian University of Health Sciences, 44307 Kaunas, Lithuania; aispra@gmail.com; 16 School of Psychology, Queen’s University Belfast, Belfast BT7 1NN, UK; lharper141@gmail.com; 17 Department of Psychology, University of Oslo, 0315 Oslo, Norway; dr.hessen@gmail.com

**Keywords:** clinical neuropsychology, working conditions, healthcare, European study

## Abstract

This study analyzed aspects of the work of clinical neuropsychologists across Europe. There are no published comparisons between European countries regarding the nature of clinical neuropsychologists’ work. Forty-one national psychological and neuropsychological societies were approached, of which 31 (76%) responded. Data from seven countries with less than 10 neuropsychologists were excluded. A license is required to practice clinical neuropsychology in 50% of the countries. Clinical neuropsychologists work independently in 62.5%. Diagnostic/assessment work is the most frequently reported activity (54%). Most neuropsychologists work in public hospitals, followed by health centers. Adult neuropsychology was the most frequent area of activity. Services in public institutions are covered by public entities (45.8%), or by a combination of patient funds and public entities (29.2%) and only 4.2% by the patient; whereas services in private institutions are covered by the patient (26.1%) and the combination of patient, public entities (21.7%) or patient and private entities (17.4%). The data suggest that the number of neuropsychologists working across European countries is considerably low in comparison to other medical professionals. The results of the survey identified similar aspects of neuropsychologists’ work, despite variations in terms of reimbursement and mechanisms, reflecting economic and healthcare differences. Estimates on the number of clinical neuropsychologists suggest insufficient access to neuropsychological services.

## 1. Introduction

Neuropsychology is a specialized domain of psychology that is focused on the multiple relationships between cognitive and affective functioning, the personality and behavior of the individual, and brain functioning. Neuropsychology combines knowledge from neurology and psychology with scientific neuropsychology focusing on the study of brain functions and clinical neuropsychologists trying to help people who have suffered brain damage (e.g., stroke, consequences of a brain tumor, traumatic brain injury, oxygen deficiency, congenital brain damage, dementia, infections of the brain). Difficulties in planning tasks of everyday life or making far-reaching decisions are typical; a lack of control is often observed in complex situations. Some of those affected underestimate their disorders, and others cannot cope with their disabilities. Many patients are unable to maintain their previous social contacts and are socially isolated, often leading to depression and anxiety.

Clinical neuropsychology includes, on the one hand, an exact diagnosis of the consequences of brain damage based on the patient’s medical history and the results of medical examinations (especially CT and MRI) and, on the other hand, the treatment of these deficits. A neuropsychological examination is usually an intensive examination of cognitive and emotional affective functions. It includes for example the investigation of attention, memory, perception (e.g., acoustic; tactile; olfactory; visual, including visual field deficits; agnosia; neglect), language (aphasia), emotions, executive functions and behavior, and disease insight (anosognosia). Based on the results of these diagnostics, an individually planned therapy then takes place with the aim of eliminating or reducing the existing problems as much as possible. A distinction is made here between compensatory techniques (compensation of the disability with methods such as the use of memory functions of the smartphone in case of forgetfulness) and restitution techniques, which focus on the restoration of deficient brain functions (e.g., attention training with exercises or rebuilding of speech, reading, writing, and arithmetic with constant exercise material). Emotional problems such as social fears or depression are often the result of severe head injuries; here, too, it is the clinical neuropsychologist’s job to deal with these worries. Depending on the type of neurological disorder, such neuropsychological therapy can take place up to five times a week; some patients also benefit from years of treatment with low-frequency meetings, whereby several exercises can also be carried out at home (e.g., with the help of computer programs). The goals are, among other things, not only the restoration of an affected person’s ability to work but also an increase in the quality of life, which is usually significantly restricted.

Within clinical neuropsychology exist several subspecialties. While (a) adult neuropsychology investigates and treats grown-up patients, (b) paediatric and child neuropsychology works with children who have suffered brain damage. Dementia is a main field in (c) geriatric neuropsychology. A forensic neuropsychologist expert (d) presents the results of neuropsychological assessment to specific juristic questions posed by attorneys and courts. Educational neuropsychology (e) transfers principles of brain functions to the education and learning of children.

Clinical neuropsychologists are applied psychologists with training and expertise in brain–behavior relationships [1]. The practice of clinical neuropsychology involves extensive knowledge about clinical interviews and the use of neuropsychological assessment methods for diagnostic and prognostic purposes. This information can also be used for planning, implementation, and evaluation of individual-specific interventions [2]. Reaching adequate competency in the field of clinical neuropsychology requires advanced knowledge of psychological principles and theory, psychological interventions, neuroanatomy, neurological disorders, psychopharmacology, neuroscience, psychometrics, and neuropsychological assessment.

Over the last few decades, many European countries have experienced an increasing demand for neuropsychological services. This development has occurred in parallel with an increased awareness of the huge contribution of brain disorders to the total morbidity burden in Europe. For example, Olesen and Leonardi [3] estimated that 23% of years of lost healthy life and 50% of years of life lived with disabilities are caused by brain diseases. The economic costs of brain disorders are correspondingly large, constituting 24% of the total direct healthcare expenditure in Europe in 2010 [4].

A recent survey from the United States [5] revealed that clinical neuropsychologists are typically involved in integrated healthcare institutions, in which they fulfill a variety of roles and provide a number of services within interdisciplinary settings that require enhanced communication skills and collaborative decision-making [6]. Similar information regarding the nature of clinical neuropsychological services, the role of clinical neuropsychologists in healthcare, and methods of compensation do not exist for many European countries.

The European Federation of Psychologists’ Associations (EFPA) established a Standing Committee on Clinical Neuropsychology (SCCN) in 2016. The motivation for this committee was to assess the field of clinical neuropsychology across Europe, namely to collect information on training in neuropsychology and the legal and professional status of clinical neuropsychologists in European countries and to make recommendations for the development of the discipline.

Pursuing its goals, the SCCN developed an online survey to obtain information on the role of clinical neuropsychology in healthcare systems and public access to neuropsychological services (diagnostics and treatment) across Europe. This specifically was examined with the aim of developing a European benchmark for a suitable qualification in clinical neuropsychology, which would be helpful in reducing the heterogeneity in training programs and practice standards between countries. The long-term goal of these papers was to pave the way toward a EuroPsy specialist certificate model for clinical neuropsychologists, thus raising the European standards for training and practice in the field.

This article examines different aspects of the work of clinical neuropsychologists in Europe, namely in respect to its functional content, institutions/settings in which neuropsychologists work, target populations, and financial mechanisms that pay for the services of clinical neuropsychologists. Thus, here we report the results of the survey regarding the role and aspects of working conditions of clinical neuropsychologists in the healthcare system in European countries. These results, together with the previously published results by the SCCN, set the basis for creating guidelines to optimally prepare and certify psychologists for their work in this specialized field across Europe.

Specifically, the aims of the present study were (1) to present information on the current work environment/conditions of clinical neuropsychologists across Europe (target populations, work settings, licensure status, and type of services they offer within their work settings); (2) to analyze information about the level of autonomy of clinical neuropsychologists providing services independently from medicine and other disciplines in relation to legal status; and (3) to present information regarding sources of reimbursement for clinical neuropsychological services.

## 2. Methods

An online survey was prepared in 2016/17 and distributed across Europe by the EFPA’s Standing Committee on clinical neuropsychology in 2017/18. Before distributing the survey, a pilot study was conducted with five respondents to ensure high practicality and clarity of the questions. After final corrections (e.g., improvement of poorly understandable questions and deletion of superfluous questions), the survey included 36 questions in English. Further details on the survey preparation and a full copy can be found in the works of Hokkanen et al. [7,8].

A link to the survey was disseminated via e-mail to the presidents of all EFPA member associations (*n* = 36 national psychological associations) and the chairpersons of all member organizations (*n* = 16 national neuropsychological societies) of the Federation of European Societies of Neuropsychology (FESN). All countries with an FESN member organization also had an EFPA member association. Representatives of five European countries that are not members of either organization were also invited to participate. Therefore, a total of 41 countries were invited to participate in the survey. In some countries, both the representative of the EFPA and the representative of the FESN responded. In such cases, the data were compared and, if necessary, adjusted [7]. Two reminders were sent when there was no response.

Representatives from 31 (76%) of the countries responded; 16 (52%) were presidents of the organization, and the others were secretaries or board members within their organization. All had a background in psychology or neuropsychology (see Table 1). Countries in which respondents reported 10 or fewer neuropsychologists (Cyprus, Latvia, Lichtenstein, Lithuania, and Serbia) were excluded. With fewer than 10 neuropsychologists in a country, the authors assumed that there was no specialized vocational training and no uniform field of work to be analyzed. Responses from Hungary and San Marino were only a general description of their situation without sufficient detail to be used in the analyses and were also excluded. The data from the remaining 24 countries (Austria, Belgium, Croatia, Czech Republic, Denmark, Estonia, Finland, France, Germany, Greece, Iceland, Ireland, Italy, Luxembourg, the Netherlands, Norway, Poland, Portugal, Russia, Spain, Sweden, Switzerland, Turkey, and the United Kingdom) were analyzed. Since we received very precise data from Cyprus and Lithuania, this information is included in some of the tables, but it is not included in the analysis.

The following information was collected for each country: respondent’s country, the responding organization, names of the responding members, their contact information, and the respondent’s position within the organization. Of these 24 respondents, 9 (37%) were the heads of the organization. Most of the others were board members or secretaries within the organization, with significant experience of working as a clinical neuropsychologist and knowledge of neuropsychological services in their country. The majority of the (neuro)psychological societies had access to existing registries and other data, so that precise information could be provided.

For the purpose of the survey, a clinical neuropsychologist was defined as an adequately trained professional who spends 50% or more of his/her time in one or more of the following activities: neuropsychological clinical practice, patient work in clinical neuropsychological assessment, and neuropsychological rehabilitation. The activities were not to be limited to research or teaching only. Levels of university education were defined referring to the Bologna three-cycle model: bachelor’s, master’s, and doctorate degree (European Commission/EACEA/Eurydice, 2015). If the national degrees differed from this model, clarification was requested.

## 3. Results

**Figure 1:**
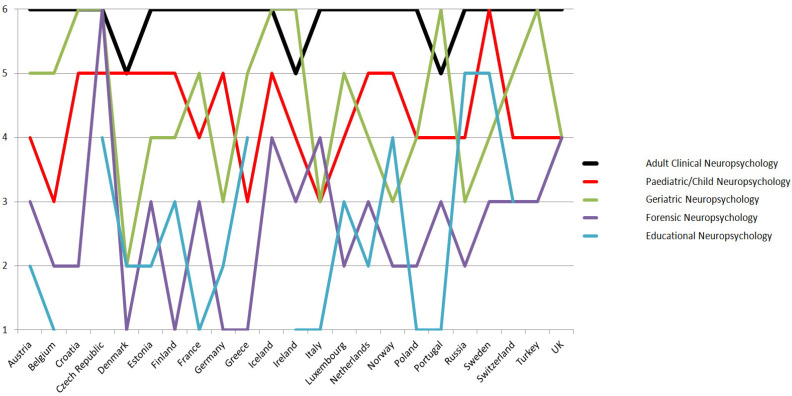
Areas of practice listed from least common (1) to most common (6). If fields of work were considered equal, the same value could be assigned multiple times. The choice “other” is not included in this figure; data from Spain are missing.

The results relate to the 24 European countries listed above. Respondents were requested to rank the following areas of practice/target populations of clinical neuropsychology in their countries from the least common to the most common: (a) adult clinical neuropsychology, (b) pediatric/child neuropsychology, (c) geriatric neuropsychology, (d) forensic neuropsychology, (e) educational neuropsychology, and (f) other (e.g., special examinations). Results were ordered from the least common (1) to the most common (6), so that the higher the value, the more common is the field of practice and target population (see Figure 1). Based on responses from 23 countries (1 missing), adult clinical neuropsychology was the most common area of practice/target population (*M* = 5.87, *SD* = 0.41), followed by geriatric neuropsychology (*M* = 4.52, *SD* = 1.20), pediatric/child neuropsychology (*M* = 4.43, *SD* = 0.84), forensic neuropsychology (*M* = 2.65, *SD* = 1.19), and educational neuropsychology (*M* = 2.63, *SD* = 1.50).

When asked in what public settings clinical neuropsychology services are provided, the majority indicated public hospitals (91.7%), healthcare centers (75.0%), rehabilitation (73.9%), and reintegration/occupational training centers (50.0%), for both inpatients and outpatients.

Respondents were asked whether a legal licensure/registration was required in order to practice clinical neuropsychology in the respondents’ country. Half of the respondents (*n* = 12, 50%) said that they did not need a clinical neuropsychology licensure/registration to practice, whereas only three (12.5%) respondents reported that either there is a law protecting the title of clinical neuropsychologist or that a license/registration to practice clinical neuropsychology is required in their countries (see Table 2 for details). However, a general license as a clinical psychologist or psychotherapist is often sufficient to also offer neuropsychological services, and in some cases, the requirements for a certificate as a neuropsychologist are not adequately specified.

When asked whether clinical neuropsychologists overall work independently or are subordinate to others, nine (37.5%) of the respondents answered that clinical neuropsychologists are subordinate to other professions in their countries (e.g., medical doctors), meaning that in most countries (*n* = 15; 62.5%) they work as independent professionals (Table 2). When asked specifically regarding diagnoses based on DSM or ICD criteria, 15 (65.2%) of the countries surveyed indicated that clinical neuropsychologists are authorized to make their own independent diagnoses, but 8 (34.8%) countries did not (Estonia, Finland, France, Ireland, Italy, Luxembourg, Poland, Russia, one m.d.). In 22 countries (91.7%), clinical neuropsychologists conduct independent neuropsychological rehabilitation and psychological treatments in their healthcare systems; only 2 (8.3%) of the 24 respondents gave a negative answer. In 19 countries (79.2%), clinical neuropsychologists could be the head of multidisciplinary departments (e.g., mental health or rehabilitation departments), divisions, or clinics within their public healthcare system, while this was not possible in 5 countries (20.8%; Estonia, Italy, Luxembourg, Russia, Turkey).

The length of the training varies widely between one and five years. For some countries, no clear information can be given, because, e.g., training in neuropsychology is often included in the curriculum to become a psychotherapist; i.e., anyone who is certified can also conduct neuropsychology (e.g., France). For example, there is no universal formalized training in Iceland. People get their specialization based on education (which can be Ph.D. or M.D.) and subsequent work/supervision and added education (e.g., conferences)—all those qualifications are then evaluated by a special qualification board which then issues a specialist license. In Portugal, the 48 months means a mean duration, comprising both training and supervised practice (see Table 2).

Respondents from 13 (54.2%) of the 24 European countries included in the analyses indicated that clinical neuropsychologists mostly do diagnostic/assessment work, while 11 (45.8%) reported that neuropsychologists in their countries are doing about the same amount of diagnostic/assessment and rehabilitation/treatment work. None of the respondents stated that neuropsychologists are mostly involved in treatment/rehabilitation work.

The respondents were asked to rank the activities of clinical neuropsychologists in their countries from the least common (1) to the most common (6), based on the type of practice. Neuropsychological evaluation and assessment were ranked as the most common activity (*M* = 5.87, *SD* = 0.46) based on the ratings from 23/24 responding countries. Neuropsychological rehabilitation/intervention/therapy was the second most frequent activity (*M* = 4.78, *SD* = 0.80), followed by neuropsychological consultation (*M* = 3.96, *SD* = 1.30), and neuropsychological research (*M* = 3.90, *SD* =1.00); only two countries chose the category “other activities” (*M* = 2.00).

Respondents were asked to respond to questions relating to the usual source of compensation for the costs of clinical neuropsychological services. The possible responses were (a) entirely by the patient; (b) entirely by public entities (i.e., governmentally funded medical systems, including when patients/clients pay up front but are fully reimbursed later); (c) entirely by private entities (i.e., private insurance; including when patients/clients pay up front, but are fully reimbursed later); (d) the patient pays a part, whereas public entities pay the remaining; and (e) the patient pays a part, whereas private entities pay the remaining. The same question was asked regarding services provided within private institutions in the countries surveyed.

**Figure 2:**
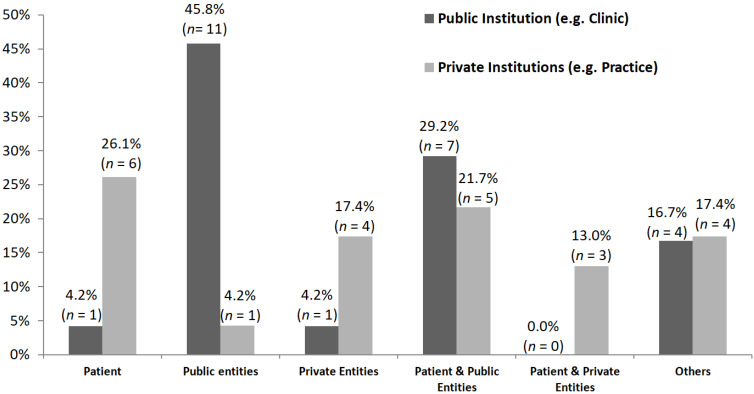
The graphic shows who pays for neuropsychological treatment in a public institution (*n* = 24 respondents, dark gray), e.g., a clinic, or in a private institution (*n* = 23 answers, light gray), e.g., an outpatient practice.

Nearly half of the respondents indicated that services in public institutions are covered by public entities (45.8%), with many indicating that services in public institutions are covered by a combination of patient funds and public entities (29.2%) and only 4.2% indicating that services in public institutions are covered by the patient. The respondents indicated that services in private institutions are often covered by the patient (26.1%) and the combination of patient and public entities (21.7%), and 17.4% chose the option “other”. For more detailed information, see Table 3 and Figure 2.

Finally, respondents were asked to give an estimate of the number of trained clinical neuropsychologists working in their country. The number varied considerably across European countries. An estimate of the number of clinical neuropsychologists ranged from 0.05 to a maximum of 10 per 100,000 inhabitants (mean 3.0 per 100,000; estimates based only on countries with more than 10 neuropsychologists). These estimates were then compared to estimates of relevant medical specialists (see notes of Table 4 for sources). The number of psychiatrists among the European countries included in the present study varied between 1.6 and 48.0 per 100,000 inhabitants (with an average of 17.6 psychiatrists per 100,000). The number of neurologists varied between 1.0 and 13.0 per 100,000 (with an average of 5.4 neurologists per 100,000). The healthcare budgets in these countries varied between 3.9% and 10.5% of the national GDP (with an average of 7.7%).

## 4. Discussion

Neuropsychologists work in multiple clinical and health-related contexts, delivering services to populations throughout the life span with relevant interventions to problems ranging from early neurodevelopment problems to neurodegenerative pathologies. The aim of the study was to explore the working environment and conditions of clinical neuropsychologists throughout Europe. Out of the 31 countries that responded to the survey, 24 met the inclusion criteria and were entered into the analyses.

This study has some limitations. Primarily, not all European countries could be analyzed, and in some of them, only very few neuropsychologists are active. Moreover, some of the data are based on estimates, although mainly obtained from psychological societies. The number of trained clinical neuropsychologists in a country was estimated without specification of how these estimates were derived. The variability in the number of neuropsychologists in different countries may reflect true differences in number across countries or the different ways these numbers were estimated. Despite these shortcomings, this study is the first to present data on the role and aspects of working conditions of neuropsychologists in Europe.

In the present study, we found several similarities in work settings, level of autonomy, and most common areas of practice across Europe, but some differences as well, mostly pertaining to licensure/registration requirements and sources of payment for neuropsychological services provided.

A relevant finding was that clinical neuropsychologists work with a high level of autonomy in most countries. In about two-thirds of the countries, they work independently, can head a multidisciplinary department, and are authorized to make independent DSM or ICD diagnoses in their healthcare system. Still, less than half of the respondents reported that a specific license is required to practice clinical neuropsychology in their countries. This may mean that there are no systems in place to regulate professional practice in this field, which may jeopardize the quality of the services provided. In countries where the title of a clinical neuropsychologist was reported to be protected by law, the level of independence seemed highest. If the regulation was performed using other national licensing instruments, the level of independence was more open to variation.

In terms of work activities, about half of the respondents answered that clinical neuropsychologists do mostly diagnostic/assessment work, while the other half reported approximately equal involvement in diagnostic/assessment and rehabilitation/treatment work. The results are in accordance with data from the United States, showing that neuropsychologists spend most of their professional time conducting neuropsychological assessments [20], and the length of time reported for evaluations appears to be increasing over time [21]. A recent paper by Sweet et al. [22] involving a U.S. sample of 1677 doctoral-level practitioners, the largest sample surveyed within the specialty of clinical neuropsychology so far, found that the mean hours necessary to complete outpatient evaluations is steadily decreasing, with these shortened evaluation times possibly reflecting negative effects of healthcare changes that may be happening in Europe as well.

Public hospitals and public rehabilitation centers were the most common occupational environments for clinical neuropsychologists, followed by health centers and public reintegration or occupational training centers. Adult clinical neuropsychology was the most common area of work, followed by geriatric, pediatric, forensic, and educational neuropsychology. Official subspecialties within clinical neuropsychology in Europe are rare, but child/pediatric subspecialties are available in some countries, and five separate subspecialties are acknowledged in Spain [7]. According to the results of Sweet et al. [22], the interest in subspecialization certifications is relatively high in the United States, with more than half of practitioners engaging in forensic neuropsychology, but neurologists remain the most important referral source, independent of working in an institution, private practice, or a combination of both and regardless of maintaining a pediatric, adult, or lifespan practice.

The payment systems for neuropsychological services are very diverse across Europe. When neuropsychological services are provided in public institutions, in most countries, the costs are covered by the government, whereas in some countries, the costs are covered by a combination of patient and public funds, with very few countries indicating that the costs remain solely on the patients themselves. When neuropsychological services are provided in private institutions (e.g., a neuropsychological practice or office), in most countries, costs are paid entirely by the patient or entirely by private companies. However, in some countries, they are covered by a combination of patient and government funding; in very few countries, they are paid entirely by the government, which can be interpreted as an acknowledged public need for these services despite the fact that they are provided privately. Some of the differences may reflect completely different social systems in Europe, as explained below.

Even in countries where neuropsychology is well established and regulated, such as in the United Kingdom, Germany, and the Nordic countries, information about clinicians in neuropsychology and their work-related activities and work settings is still scarce [23]. Most studies providing information on neuropsychologists and their activities have been conducted in the United States, which has played an important role in the development of the practice and science of clinical neuropsychology worldwide (e.g., see [24,25,26,27,28]). Although regulations vary from state to state in the United States too, it might be easier in the United States to regulate education, licensing, and activities than in Europe. For example, recent surveys showed that 91% of the clinical neuropsychologists in the United States work full-time, and 53% in research institutions only, with an annual income of approximately EUR 120,000 [21]. In contrast, 72% of Nordic neuropsychologists work full-time, mostly in hospital settings (66%), with a mean annual income of about EUR 50,000 [23]. The practice of clinical neuropsychology in other European countries is likely to be less favorable. According to a survey of Spanish neuropsychologists [29], only 28% of the respondents worked in hospitals, 51% reported to be working full-time, and the yearly income was about EUR 17,000. Of course, income must always be seen in light of the cost of living, and in the present survey, we did not ask about the income or full-time vs part-time working. Research institutes offering clinical services did not appear in the present survey. In the United States, the “models” under which payments for neuropsychologists’ services are made can vary widely, as can the methods by which their work may be recognized and rewarded by institutions [24].

Because of the different social systems, it is difficult to compare the European situation with the United States. A comparison within Europe is also difficult. Cross-country differences revealed in this survey regarding several aspects of the neuropsychologist’s work and compensation of clinical neuropsychologists most likely reflect diverse social and medical cost reimbursement systems in Europe. Historically, two different types of public healthcare have emerged, also known as the “Beveridge” and “Bismarck” systems after their founding fathers: The Beveridge systems are state, tax-funded networks of physicians’ offices and hospitals to which all residents have access. They were first established in Great Britain (United Kingdom) after World War II on the basis of the report of a parliamentary commission led by Lord Beveridge. Comparable health systems can be found, for example, in the Scandinavian countries, Canada, Italy, and Spain. The “Bismarck” systems are social health insurances that are financed by social contributions of the insured and their employers. They have their historical roots in the statutory health insurance introduced by Bismarck in Germany, in 1883. There are three different basic directions: regional or centralized insurance (e.g., in France, Poland, and the Czech Republic), company or professional and regional compulsory insurance (e.g., in Belgium, Japan, Austria), and structured system with free choice of health insurance provider and box office competition (in Germany, the Netherlands, and Switzerland).

In fact, in several European countries, medical care (which would include clinical neuropsychology), is mainly financed by the government (e.g., the United Kingdom, Lithuania, Portugal, Norway), whereas in other countries, it is almost exclusively supported by social security contributions (e.g., Belgium, Germany, Luxembourg); often there are mixed models as well (e.g., Estonia, Greece, Italy, Austria). For example, while there is only one basic insurance medical scheme in Switzerland, there is a two-class medical compensation scheme with private and government insurance in Germany. In France, in addition to general health insurance, there are many small special health insurance companies. Finally, the economic situation of a country may also be associated with variations in salary and the cost of healthcare across Europe.

## 5. Conclusions

In the last 30 years, clinical neuropsychology has developed from a marginal clinical discipline to a discipline that is well integrated into the healthcare system in several European countries. In most of these countries, the practice of clinical neuropsychology is now expected to be conducted only by trained specialists. As knowledge about the relationship between the brain and behavior expands, clinical neuropsychologists are in a unique position to provide crucial diagnostic and other information for the optimal treatment of patients with brain dysfunction. Indeed, the need for clinical neuropsychological services has already been demonstrated in several fields of practice: neurodevelopment and learning disorders [30], dementia [31], stroke [32,33], TBI [34], psychiatric and forensic cases [35], and epilepsy [36], as well as in a number of vascular, inflammatory, and autoimmune diseases. However, despite this contribution, there is sufficient evidence of the incremental value of neuropsychological assessment in clinical practice, both for diagnostic classification and prediction of long-term daily-life outcomes in patients across the lifespan [37].

Despite the increasing need for specialists in clinical neuropsychology, estimates of their number are relatively low in each of the European countries included in the present study. When compared to the number of relevant medical specialists in each of these countries, specifically psychiatrists and neurologists, we found considerably greater variability across countries in the numbers of clinical neuropsychologists per 100,000 inhabitants, as well as a smaller mean number of clinical neuropsychologists than psychiatrists and neurologists. Interestingly, but not surprisingly, many countries with low relative health costs also have the lowest numbers of clinical neuropsychologists. However, the countries with the highest social benefits do not necessarily have the highest number of neuropsychologists. For example, Denmark, which is in the middle range in its health budget, has one of the highest proportions of neuropsychologists (about 10 per 100,000). Germany and the Netherlands, which are at the top of social spending, have only about one neuropsychologist per 100,000 people.

As clinical neuropsychology has moved beyond mere diagnostic and descriptive work into rehabilitation and treatment, the growing need for such services has become evident, regardless of the lack of rigorous and updated estimates on the need for clinical neuropsychologists in Europe. To give an example, an older article by Kasten, Eder, Robra, and Sabel [38] found an incidence of around 500,000 patients with brain damage in Germany. After subtracting deceased, severely impaired, and only very slightly impaired patients, the article comes to a number of around 10% of all patients who would benefit from neuropsychological treatment after brain damage, i.e., around 50,000 patients per year. In Germany, there are currently only 700 certified neuropsychologists, who cannot meet this need.

Furthermore, neuropsychological services should also include maintaining cognitive health through the prevention of adverse factors and the promotion of protective and enhancing lifestyle factors beginning from early life to delaying cognitive decline in old age [39], given that cognitive health begins at conception [40]. Clinical neuropsychologists are involved in research and clinical practice with these goals in mind, yet more specialists and funds are needed to augment public awareness, lifestyle, and cognitive health in Europe.

Identification of the commonalities and differences among European countries with respect to aspects of the role and work of clinical neuropsychologists that were analyzed here may help to optimize training and practices throughout Europe, setting the highest standards both for the clinical neuropsychologists providing the services and those who benefit from these services. As Europe is growing closer together and professional migration from one country to another is becoming more frequent, it seems important to set a common ground regarding the training and roles of clinical neuropsychologists in healthcare, so that moving from one country to another is not a problem. A specialist EUROPSY certificate in clinical neuropsychology would be a step toward this goal, and the results presented in this study are relevant to informing such an endeavor.

## Figures and Tables

**Table 1:** Information about the societies of the respondents.

Country	Society
Austria	Austrian Professional Association of Psychologists
Belgium	Flemish Neuropsychological societu
Croatia	Croatian Psychological Association
Cyprus	Cyprus Psychologists’ Association
Czech Republic	Czech Neuropsychological Society
Denmark	Selskabet for Danske Neuropsykologer
Estiona	Union of Estonian Psychologists
Finland	Finnish Neuropsychological Society
France	French Federation of Psychologists and Psychology
Germany	Gesellschaft für Neuropsychologie
Greece	Hellenic Neuropsychological Society
Hungary	Neuropsychology Section of the MPT
Iceland	Icelandic Psychological Association
Ireland	Psychological Society of Ireland Division of Neuropsychology
Italy	Società Italiana di Neuropsicologia
Latvia	Latvian Professional Psychologists Association
Liechtenstein	Berufsverband der Psychologinnen und Psychologen Liechtensteins
Lithuania	Lithuanian University of Health Sciences, Neurosciences Institute
Luxembourg	Task force of the Luxemburgish Society of Psychology
Netherlands	Data taken from: Stichting PAON delegated to RINO-Groep
Norway	The Norwegian Neuropsychological Society
Poland	Polish Psychological Association
Portugal	Behavioral Neurology Section of the Portuguese Society of Neurology Ordem dos Psicólogos Portugueses
Russia	Lomonosov Moscow State University
San Marino	Ordine degli Psicologi in San Marino
Serbia	Clinic of Psychiatry, Clinical Center of Serbia, Faculty of Media and Communications, Department of Psychology
Spain	Federation of Spanish Societies of Neuropsychology
Sweden	Swedish Neuropsychological society
Switzerland	Swiss Society of Neuropsychology
Turkey	Turkish Society of Neuropsychology-NPD
United Kingdom	Division of Neuropsychology

**Table 2:** Detailed information for the individual countries about whether a law or regulation protects the title of clinical neuropsychologist, the lengths of training in months (after bachelor and master), license as a specialist, subject to other professions and permission for independent treatment.

Country	Title as Clin. Neuropsychologist Protected by Law	Length of Education (Months)	Specialist Licensure	Subordinate to Other Professions	Independent Treatment
Austria	Yes	24	Yes	No	Yes
Belgium	No	24	No	Yes	Yes
Croatia	No	No specialized training	No	No	Yes
Czech Republic	No	18	Yes	Yes	Yes
Cyprus	No	Not applicable	No	No	Yes
Denmark	No	Variable, text	No	Yes	Yes
					
Finland	No	36	No	No	Yes
France	No	Variable, see text	No	No	Yes
Germany	No	36	Yes	Yes	Yes
Greece	No	24	No	No	Yes
Iceland	No	Variable, see text	Yes	Yes	Yes
Ireland	No	Variable, see text	Yes	No	Yes
Italy	No	48	Yes	No	Yes
Luxembourg	No	12	Yes	No	No
Netherlands	Yes	48	Yes	Yes	Yes
Norway	No	60	Yes	No	Yes
Poland	No	48	No	No	Yes
Portugal	Yes	Variable, see text	Yes	No	Yes
Russia	No	55–60	No (yes for clinical psychologists; clin. neuropsy. is a variant of clin. psychology)	No	Yes
Spain	No	36	No	Yes	Yes
Sweden	No	60	Yes	No	Yes
Switzerland	No	Variable, see text	No	Yes	No
Turkey	No	Variable, see text	No	No	Yes
United Kingdom	No	24	No	Yes	Yes

**Table 3:** Detailed information about payment for clinical neuropsychology.

Country	Public Institutions (e.g., Clinic, Hospital)	Private Institutions (e.g., Practice)
Austria	Entirely by public entities	Others (depends on the underlying pathology and includes private/public/mixed)
Belgium	Entirely by the patient/client	The patient pays a part, whereas public entities pay the remaining
Croatia	Entirely by public entities	The patient pays a part, whereas private entities pay the remaining
Cyprus	The patient pays a part, whereas public entities pay the remaining	Others
Czech Republic	Entirely by public entities	Others (depends on the underlying pathology and includes private/public/mixed)
Denmark	Entirely by private entities	m.d.
Estonia	The patient pays a part, whereas public entities pay the remaining	Others
Finland	Entirely by public entities	Entirely by private entities
France	Others (depends on the underlying pathology and includes private/public/mixed)	The patient pays a part, whereas private entities pay the remaining
Germany	Entirely by public entities	Entirely by public entities
Greece	The patient pays a part, whereas public entities pay the remaining	Entirely by the patient
Iceland	The patient pays a part, whereas public entities pay the remaining	Entirely by the patient
Ireland	Entirely by public entities	Entirely by the patient
Italy	Entirely by public entities	Entirely by the patient
Latvia	m.d.	m.d.
Liechtenstein	Others	Others
Lithuania	Inpatient services are paid for by public entities, but outpatient services might be governmental, paid for by public entities	Entirely by the patient
Luxembourg	Others	Entirely by the patient
Norway	The patient pays a part, whereas public entities pay the remaining	The patient pays approximately 4% of the total cost, whereas public entities pay the remaining, approximately 96%
Poland	Entirely by public entities	Entirely by private entities
Portugal	The patient pays a part, whereas public entities pay the remaining	The patient pays a part, whereas private entities pay the remaining
Russia	In public clinics, it is paid for entirely by public entities; in private clinics, it can be paid for by public entities and/or others	Entirely by the patient
Serbia	Entirely by public entities	Entirely by the patient
Spain	Entirely by public entities	Entirely by private entities
Sweden	The patient pays a part, maximum EUR 30, whereas public entities pay the remaining	The patient pays a part, maximum EUR 30, whereas public entities pay the remaining
Switzerland	The patient pays a part, whereas public entities pay the remaining	Entirely by the patient
the Netherlands	Others (depends on the underlying pathology and includes from private/public/mixed)	Others (depends on the underlying pathology and includes private/public/mixed)
Turkey	The patient pays a part, whereas public entities pay the remaining	The patient pays a part, whereas public entities pay the remaining
UK	Entirely by public entities	Entirely by public entities

**Table 4:** Number of psychiatrists, neurologists, and neuropsychologists working in the healthcare sector (the data for the number of neuropsychologists were obtained from the (neuro)psychological societies, and the other information comes from different sources (see notes); Mio = million).

Country	Psychiatrists per 100,000	Neurologists per 100,000	Neuropsychologists	Neuropsychologists per 100,000	
Austria	16.9	9.6	650	7.4	(650:8.8 Mio)
Belgium	20.1	6.5	200	1.8	(200:11.4 Mio)
Croatia	11.1	6.2	15	0.4	(15:4.2 Mio)
Czech Republic	12.4	6.3	20	0.2	(20:10.6 Mio)
Cyprus	11.6	Unavailable data	3	0.3	(4:1.0 Mio)
Denmark	17.0	3.0	550	9.6	(550:5.7 Mio)
Estonia	16.2	13.0	23	1.8	(23:1.3 Mio)
Finland	23.6	6.3	250	4.5	(250:5.5 Mio)
France	20.9	3.6	5000	7.7	(5.000:65.0 Mio) Mio)
Germany	13.2	5.5	700	0.9	(700:82.1 Mio)
Greece	5.8	10.9	60	0.5	(60:11.2 Mio)
Iceland	26.0	6.5	13	3.5	(14:0.4 Mio)
Ireland	16.0	0.4	40	0.9	(40:4.7 Mio)
Italy	6.0	5.9	2000	3.4	(2.000:59.4 Mio)
Luxembourg	17.8	4.6	50	10.0	(50:0.5 Mio)
Netherlands	20.9	4.7	150	0.9	(150:17.0 Mio)
Norway	48.0	7.7	330	6.2	(330:5.3 Mio)
Poland	24.2	7.0	100	0.3	(100:38.2 Mio)
Portugal	11.0	3.4	150	1.5	(150:1.3 Mio)
Russia	8.5	1.3	600	5.8	(600:104.0 Mio)
Spain	3.6	4.4	10	2.2	(1.000:46.3 Mio)
Sweden	20.9	4.0	1000	5.1	(500:9.9 Mio)
Switzerland	44.0	5.2	500	3.5	(300:8.5 Mio)
Turkey	1.6	2.0	300	0.4	(40:9.8 Mio)
United Kingdom	17.1	1.0	600	1.0	(600:66.2 Mio)
Average	17.6	5.4	400	3.1	

Notes. Data were gathered from sources [9,10,11,12,13,14,15,16,17,18,19].

## Data Availability

The original data can be obtained from the first author on request.

## References

[1] Barth J.T., Pliskin N., Axelrod B., Faust D., Fisher J., Harley J.P., Heilbronner R., Larrabee G., Puente A., Ricker J. (2003). Introduction to the NAN 2001 Definition of a clinical neuropsychologist-NAN Policy and Planning Committee. Arch. Clin. Neuropsychol..

[2] EFPA Standing Committee on Clinical Neuropsychology (2018). European Definition of Clinical Neuropsychology. http://clinneuropsy.efpa.eu.

[3] Olesen J., Leonardi M. (2003). The burden of brain diseases in Europe. Eur. J. Neurol..

[4] Olesen J., Gustavsson A., Svensson M., Wittchen H., Jönsson B. (2012). The economic cost of brain disorders in Europe. Eur. J. Neurol..

[5] Kubu C.S., Ready R.E., Festa J.R., Roper B.L., Pliskin N.H. (2016). The Times They Are a Changin: Neuropsychology and Integrated Care Teams. Clin. Neuropsychol..

[6] Roper B.L., Block C.K., Osborn K., Ready R.E. (2018). Education and Training for clinical neuropsychologists in Integrated Care Settings. Arch. Clin. Neuropsychol..

[7] Hokkanen L., Lettner S., Barbosa F., Constantinou M., Harper L., Kasten E., Mondini S., Persson B., Varako N., Hessen E. (2019). Training Models and Status of clinical neuropsychologists in Europe: Results of a Survey on 30 Countries. Clin. Neuropsychol..

[8] Hokkanen L., Barbosa F., Ponchel A., Constantinou M., Kosmidis M.H., Varako N., Kasten E., Mondini S., Lettner S., Baker G. (2020). Clinical neuropsychology as a Specialist Profession in European Health Care: Developing a Benchmark for Training Standards and Competencies Using the Europsy Model?. Front. Psychol..

[9] Global Health Observatory Data Repository. https://apps.who.int/gho/data/node.main.MHHR?lang=en.

[10] Mental Health: How Many Psychiatrists in the EU?. https://ec.europa.eu/eurostat/web/products-eurostat-news/-/EDN-20171010-1.

[11] Effectifs en Neurologie France 2018 | Statista. https://fr.statista.com/statistiques/520389/nombre-medecins-neurologie-region-france/.

[12] Luxembourg in Top 10 by Number of Psychiatrists. https://luxtimes.lu/luxembourg/35837-luxembourg-in-top-10-by-number-of-psychiatrists.

[13] European Region (EUR). https://www.who.int/mental_health/evidence/Country_profiles_Europe.pdf.

[14] de Falco F.A., Inzitari D. (2013). Need for neurology specialists to be dedicated to hospital care in Italy. Neurol. Sci..

[15] van Drunen P., van Strien P.J. (1999). Psychology in The Netherlands: Recent trends and current situation. Eur. Psychol..

[16] Grisold W., Galvin R., Lisnic V., Lopes Lima J., Mueller E., Oberndorfer S., Vodusek D.B. (2007). One Europe, one neurologist?. Eur. J. Neurol..

[17] Struhal W., Sellner J., Lisnic V., Vécsei L., Müller E., Grisold W. (2011). Neurology residency training in Europe—The current situation. Eur. J. Neurol..

[18] (2019). What’s happening in Neurology^®^ Clinical Practice. Neurology.

[19] Data about Health Systems are Taken from. https://www.missoc.org/missoc-database/comparative-tables/.

[20] Rabin L.A., Barr W.B., Burton L.A. (2005). Assessment practices of clinical neuropsychologists in the United States and Canada: A survey of INS, NAN, and APA Division 40 members. Arch. Clin. Neuropsychol..

[21] Sweet J.J., Benson L.M., Nelson N.W., Moberg P.J. (2015). The American Academy of clinical neuropsychology, National Academy of Neuropsychology, and Society for clinical neuropsychology (APA Division 40) 2015 TCN professional practice and ‘salary survey’: Professional practices, beliefs, and incomes of US neuropsychologists. Clin. Neuropsychol..

[22] Sweet J., Klipfel K.M., Nelson N.W., Moberg P.J. (2020). Professional practices, beliefs, and incomes of U.S. neuropsychologists: The AACN 2021, NAN, SCN 2020 practice and “salary survey”. Clin. Neuropsychol..

[23] Norup A., Egeland J., Løvstad M., Nybo T., Persson B.A., Rivera D., Arango-Lasprilla J.C. (2017). Education, training, and practice among Nordic neuropsychologists. Results from a professional practices survey. Clin. Neuropsychol..

[24] Grote C.L., Butts A.M., Bodin D. (2016). Education, training and practice of clinical neuropsychologists in the United States of America. Clin. Neuropsychol..

[25] Kumar J.K., Sadasivan A. (2016). Neuropsychology in India. Clin. Neuropsychol..

[26] Janzen L.A., Guger S. (2016). Clinical neuropsychology practice and training in Canada. Clin. Neuropsychol..

[27] Poonsford J. (2016). The practice of clinical neuropsychology in Australia. Clin. Neuropsychol..

[28] Vakil E., Hoofien D. (2016). Clinical neuropsychology in Israel: History, training, practice and future challenges. Clin. Neuropsychol..

[29] Olabarrieta-Landa L., Caracuel A., Pérez-García M., Panyavin I., Morlett-Paredes A., Arango-Lasprilla J.C. (2016). The profession of neuropsychology in Spain: Results of a national survey. Clin. Neuropsychol..

[30] Silver C.H., Blackburn L.B., Arffa S., Barth J.T., Bush S.S., Koffler S.P., Moser R.S. (2006). The importance of neuropsychological assessment for the evaluation of childhood learning disorders: NAN Policy and Planning Committee. Arch. Clin. Neuropsychol..

[31] Maciel P.A.G., Dantas R.D.S.A., Bastos J.E.P., Sant A.L., de Oliveira R.T.P., Batista H.M.T. (2018). The Importance of Neuropsychological Assessment in the Diagnosis of Dementia in the Elderly. Amadeus Int. Multidiscip. J..

[32] Broomfield N.M., Kneebone I.I., Laidlaw K. (2014). Neuropsychological (Mood and Cognition) Consequences of Stroke. Clin. Psychol. Forum.

[33] Watson P.A., Gignac G.E., Weinborn M., Green S., Pestell C.A. (2020). Meta-Analysis of Neuropsychological Predictors of Outcome Following Stroke and Other Non-Traumatic Acquired Brain Injuries in Adults. Neuropsychol. Rev..

[34] Shany-Ur T., Bloch A., Salomon-Shushan T., Bar-Lev N., Sharoni L., Hoofien D. (2020). Efficacy of Postacute Neuropsychological Rehabilitation for Patients with Acquired Brain Injuries is Maintained in the Long-Term. J. Int. Neuropsychol. Soc..

[35] Schultz I.Z., Sepehry A.A., Greer S. (2018). Beyond Traumatic Brain Injury: Advancing Forensic Neuropsychological Assessment. Psychol. Inj. Law.

[36] Wilson S.J., Baxendale S., Barr M., Hammed S., Langfitt J., Samson S., Watanabe M., Baker G.A., Helmstadter C., Hermann B.P. (2015). Indications and expectations for neuropsychological assessment in routine epilepsy care: Report of the ILAE Neuropsychology Task Force, Diagnostic Methods Commission, 2013–2017. Epilepsia.

[37] Donders J. (2020). The incremental value of neuropsychological assessment: A critical review. Clin. Neuropsychol..

[38] Kasten E., Eder R., Robra B.-P., Sabel B.A. (1997). Requirement for out-patient neuropsychological treatment. Z. Für Neuropsychol..

[39] Livingston G., Huntley J., Sommerlad A., Ames D., Ballard C., Banerjee S., Costafreda S.G. (2020). Dementia prevention, intervention, and care: 2020 report of the Lancet Commission. Lancet.

[40] Barnett J.H., Hachinski V., Blackwell A.D. (2013). Cognitive health begins at conception: Addressing dementia as a lifelong and preventable condition. BMC Med..

